# Pharmacotherapy of Anxiety Disorders: Current and Emerging Treatment Options

**DOI:** 10.3389/fpsyt.2020.595584

**Published:** 2020-12-23

**Authors:** Amir Garakani, James W. Murrough, Rafael C. Freire, Robyn P. Thom, Kaitlyn Larkin, Frank D. Buono, Dan V. Iosifescu

**Affiliations:** ^1^Department of Psychiatry, Icahn School of Medicine at Mount Sinai, New York, NY, United States; ^2^Silver Hill Hospital, New Canaan, CT, United States; ^3^Department of Psychiatry, Yale University School of Medicine, New Haven, CT, United States; ^4^Department of Neuroscience, Icahn School of Medicine at Mount Sinai, New York, NY, United States; ^5^Department of Psychiatry and Centre for Neuroscience Studies, Queen's University, Kingston, ON, Canada; ^6^Department of Psychiatry, Massachusetts General Hospital, Harvard Medical School, Boston, MA, United States; ^7^Clinical Research Division, Nathan Kline Institute for Psychiatric Research, Orangeburg, NY, United States; ^8^Department of Psychiatry, New York University School of Medicine, New York, NY, United States

**Keywords:** anxiolytic, phobia, panic, agoraphobia, psychopharmacology, experimental

## Abstract

Anxiety disorders are the most prevalent psychiatric disorders and a leading cause of disability. While there continues to be expansive research in posttraumatic stress disorder (PTSD), depression and schizophrenia, there is a relative dearth of novel medications under investigation for anxiety disorders. This review's first aim is to summarize current pharmacological treatments (both approved and off-label) for panic disorder (PD), generalized anxiety disorder (GAD), social anxiety disorder (SAD), and specific phobias (SP), including selective serotonin reuptake inhibitors (SSRIs), serotonin norepinephrine reuptake inhibitors (SNRIs), azapirones (e.g., buspirone), mixed antidepressants (e.g., mirtazapine), antipsychotics, antihistamines (e.g., hydroxyzine), alpha- and beta-adrenergic medications (e.g., propranolol, clonidine), and GABAergic medications (benzodiazepines, pregabalin, and gabapentin). Posttraumatic stress disorder and obsessive-compulsive disorder are excluded from this review. Second, we will review novel pharmacotherapeutic agents under investigation for the treatment of anxiety disorders in adults. The pathways and neurotransmitters reviewed include serotonergic agents, glutamate modulators, GABAergic medications, neuropeptides, neurosteroids, alpha- and beta-adrenergic agents, cannabinoids, and natural remedies. The outcome of the review reveals a lack of randomized double-blind placebo- controlled trials for anxiety disorders and few studies comparing novel treatments to existing anxiolytic agents. Although there are some recent randomized controlled trials for novel agents including neuropeptides, glutamatergic agents (such as ketamine and d-cycloserine), and cannabinoids (including cannabidiol) primarily in GAD or SAD, these trials have largely been negative, with only some promise for kava and PH94B (an inhaled neurosteroid). Overall, the progression of current and future psychopharmacology research in anxiety disorders suggests that there needs to be further expansion in research of these novel pathways and larger-scale studies of promising agents with positive results from smaller trials.

## Introduction

Anxiety disorders are the most common class of psychiatric disorders, with a lifetime prevalence in the United States of around 32%, according to the National Comorbidity Survey Replication (NCS-R) (1). Among the anxiety disorders, social anxiety disorder (SAD) and specific phobia (SP) are the most common (1). According to the World Health Organization, there are about 264 million people globally who suffer from anxiety disorders, representing a 15% increase since 2005 (2). Anxiety can lead to work and school absences and have a larger cost burden than other psychiatric disorders due to their higher prevalence (3–5). Despite this, there has been far less recent research on novel medication treatments for anxiety disorders over the past 5–10 years compared to the number of experimental drug trials on treatments for major depressive disorder (MDD), bipolar disorder, and schizophrenia (www.clinicaltrials.gov).

Part of the reason for the relative paucity of new drug compounds may be the existence of Food and Drug Administration (FDA)-approved efficacious medications and psychotherapies for anxiety disorders, as well as the perception that anxiety disorders are managed adequately with the currently available treatments. The literature, however, indicates that only 60–85% of patients with anxiety disorders respond (experience at least a 50% improvement) to current biological and psychological treatments (6). In addition, only about half of the responders achieve recovery (defined as minimal anxiety symptoms) (6). There is also evidence to suggest that patients with anxiety disorders, in particular generalized anxiety disorder (GAD) and SAD (7), have high rates of recurrence and/or experience persistent anxiety symptoms, especially if they have comorbid MDD (8). There could be several explanations for the potential refractory nature of these disorders, including misdiagnosis, poor adherence to treatment, substance use, or other comorbidities, although it does suggest that conventional treatments may not be effective for all patients and alternative pharmacotherapies should be developed (9). Unfortunately, many of the treatments that are currently being investigated are simply modifications of already approved treatments.

The nosology of anxiety disorders underwent a shift with the publication of the Fifth Edition of the Diagnostic and Statistical Manual of Mental Disorders (DSM-5) in 2013 (10). Due to questions about the phenomenological and neurobiological bases of the diseases, both posttraumatic stress disorder (PTSD) and obsessive-compulsive disorder (OCD) were removed from Anxiety Disorders section and placed into their own diagnostic classes. Selective mutism and separation anxiety disorder were also added to the Anxiety Disorders section of the DSM-5. Agoraphobia was separated from and is now a disorder distinct from panic disorder (PD). Given the extensive research done separately on OCD and PTSD and their potentially divergent neurobiochemical pathways and heritability (11), this review will focus on the following DSM-5 Anxiety Disorders: PD, GAD, SAD, and SP. For a thorough review that includes both anxiety disorders and OCD and PTSD, please see Sartori and Singewald (12). Finally, although there is strong evidence for the use of psychotherapies including cognitive behavioral therapy (CBT) and exposure therapy for anxiety disorders, as well as emerging data for the role of neurostimulation strategies such as transcranial magnetic stimulation (TMS), this review will only consider pharmacotherapies. Additionally, this review will only discuss the treatment of anxiety in adults and will exclude research on children and adolescents. Ongoing clinical studies identified on the Clinical Trials database (www.clinicaltrials.gov) will be cited by their National Clinical Trial (NCT) number.

## Current Treatments for Anxiety Disorders

### Serotonergic/Norepinephrinergic Antidepressants

The Food and Drug Administration (FDA) has approved several selective serotonin reuptake inhibitors (SSRIs) and serotonin norepinephrine reuptake inhibitors (SNRIs) for PD, GAD, and SAD. Despite these classifications, medications not approved for a condition are commonly used “off-label” in clinical practice. The European Union has similar indications for the use of SSRIs and SNRIs for the treatment of anxiety disorders as the FDA but with broader indications of SSRIs (12). See Table 1 for a list of FDA-approved and off-label medications for anxiety.

**Table 1:** Current treatments for anxiety disorders.

**Medication class**	**Mechanism of action**	**FDA approvals for anxiety disorder**	**Off-label uses**	**Therapeutic dose ranges (mg/day)**
**SSRIs:**				
Fluoxetine Sertraline Citalopram Escitalopram Paroxetine Paroxetine ER Fluvoxamine	Selective 5-HT reuptake inhibitor (20)	PD PD, SAD None GAD PD, SAD, GAD PD, SAD None	GAD, SAD GAD GAD, PD, SAD PD, SAD None GAD GAD, PD, SAD	20–60 50–200 20–40 10–20 20–60 27–75 100–300
**SNRIs:**				
Duloxetine Venlafaxine (XR) Desvenlafaxine	5-HT, NE (and DA) reuptake inhibitor (17)	GAD GAD None	PD, SAD PD, SAD GAD, PD, SAD	30–60 75–300 50–100
**TCAs:**				
Clomipramine Imipramine Desipramine Nortriptyline	NE and 5-HT reuptake inhibitor (20)	None None None None	GAD, PD, SAD GAD, PD, SAD GAD, PD, SAD GAD, PD, SAD	100–250 100–300 100–200 50–150
**MAOIs:**				
Phenelzine	MAO inhibitor (21)	None	GAD, PD, SAD	30–90
**Mixed antidepressants:**				
Mirtazapine	5-HT_2_, 5-HT_3_, α_2_, H_1_ antagonist (27)	None	Anxiety, GAD, PD, SAD	15–45
**GABAergic drugs:**				
Pregabalin Gabapentin	Unclear, may modulate Ca channels (51)	None None	GAD, SAD GAD, SAD, PD	150–600 600–2,400
**Benzodiazepines:**				
Clonazepam Alprazolam Lorazepam Chlordiazepoxide Oxazepam	GABA-A agonist (44)	PD Anxiety, PD Anxiety Anxiety Anxiety	Anxiety, GAD, PD, SAD GAD, PD, SAD GAD, PD, SAD GAD, PD, SAD GAD, PD, SAD	1–2 1–4 2–6 20–100 30–60
**Antipsychotics:**				
Trifluoperazine Olanzapine Quetiapine	D_2_ antagonist (84) D_2_, 5-HT_2_ H_1_ antagonist (85) D_2_, 5-HT_2_ H_1_ antagonist (85)	Anxiety None None	GAD, PD, SAD Anxiety, GAD Anxiety, GAD	2–6 5–15 50–300
**Beta-blockers:**				
Propranolol	β-1, β-2 antagonist (77)	None	Anxiety, PD, SAD	60–120
**Antihistamines:**				
Hydroxyzine	H_1_ antagonist (76)	Anxiety	GAD, PD, SAD	25–100
**Other anxiolytics:**				
Buspirone	5-HT_1A_ partial agonist (22)	Anxiety	GAD	15–60

*Key: 5-HT, Serotonin; AGP, Agoraphobia; DA, Dopamine; D_2_, dopamine-2 receptor; ER, XR, Extended Release; FDA, Food and Drug Administration; GAD, Generalized Anxiety Disorder; GABA, Gamma Aminobutyric Acid; H_1_, Histamine 1 receptor; MAO, Monoamine Oxidase; MAOI, Monoamine Oxidase Inhibitors; NE, Norepinephrine; PD, Panic Disorder; SSRI, Selective Serotonin Reuptake Inhibitor; SNRI, Serotonin Norepinephrine Reuptake Inhibitor; SAD, Social Anxiety Disorder; TCA, Tricyclic Antidepressants*.

Selective serotonin reuptake inhibitors and SNRIs are both first-line treatments for PD, GAD, and SAD and have been shown to be efficacious for the treatment of anxiety disorders (13–16). A recent meta-analysis reported that most SSRIs and SNRIs are more efficacious than placebo in GAD, with escitalopram and duloxetine potentially having the largest effect sizes (17). The recommended duration of treatment can vary but may be as short as 3–6 months, or up to 1–2 years or even longer. Although there may be concern about tachyphylaxis, there is limited evidence of adverse outcomes with the chronic use of SSRIs or SNRIs (18). These medications also tend to be well-tolerated, with usually manageable or short-lived adverse effects such as nausea, headache, dry mouth, diarrhea, or constipation. Sexual dysfunction tends to be a more durable and problematic adverse effect of SSRIs and SNRIs but can be managed with adjunctive treatments. There is the possibility of patients developing antidepressant-induced jitteriness or anxiety, potentially due to initial surge of serotonin, although this anxiety can be mitigated by slower titration or adjunctive use of benzodiazepines (19).

The tricyclic antidepressants (TCAs), which act as reuptake inhibitors of serotonin and norepinephrine transporters, were one of the first classes of medications used for anxiety disorders (20). Despite comparable efficacy to SSRIs, they are now less frequently prescribed due to concerns about side effects including weight gain, dry mouth, sedation, urinary hesitancy or retention, arrhythmias, and risk of mortality with overdose (20). Clomipramine and imipramine (both TCAs) are FDA-approved for PD. Monoamine oxidase inhibitors (MAOIs) are also older antidepressant medications which are now typically used only as a third-line option because of side effects and dietary restrictions. They are not FDA-approved for anxiety disorders but may be considered in patients with SAD who are non-responsive to SSRIs (21).

Buspirone, a 5-HT_1A_ partial agonist classified under the azapirones, is FDA-approved for use in anxiety, and is commonly used as an adjunctive treatment with SSRIs or SNRIs primarily for GAD (22). It is the only azapirone currently approved in the United States. A Cochrane review of buspirone for GAD found that it was superior to placebo but had a smaller effect size in GAD compared to benzodiazepines and antidepressants (22). Moreover, it was not as well-tolerated (nausea and dizziness) and less effective in those with past benzodiazepine use (22). A subsequent Cochrane review compared buspirone to placebo for PD and found buspirone to be less efficacious than placebo but the review was limited by the dearth of high-quality studies (23). Buspirone is generally dosed two to three times a day and has a gradual onset of action of around 10 days to 4 weeks. Adverse effects include nausea, dizziness, and headache, and there are reports of buspirone-induced movement disorders (24). There is also anecdotal reporting for using buspirone to offset sexual side effects from SSRIs but there are few studies offering empirical support of this practice (25, 26).

### Mixed Antidepressants

Mirtazapine has a broad pharmacological effect, with presynaptic antagonism of the alpha-2 adrenergic receptor, postsynaptic blockade of 5-HT_2_ and 5-HT_3_ receptors, and antagonism of histamine-1 (H_1_) receptors (27). Mirtazapine is FDA-approved for the treatment of MDD in adults. Its benefits include positive effects on sleep and appetite and its general safety for elderly patients, fewer drug-drug interactions, and less likelihood of sexual side effects compared to SSRIs and SNRIs. Adverse effects include weight gain and other antihistamine effects like sedation and dry mouth. There are very few clinical trials assessing mirtazapine for anxiety disorders. In PD, one small randomized controlled trial (RCT) reported that mirtazapine was comparable in efficacy to escitalopram (28). In SAD, one RCT of women showed a significant improvement in anxiety symptoms compared to placebo (29), while a subsequent study failed to show separation from placebo (30). There are no controlled studies of mirtazapine in GAD to date. Overall, in the absence of further trials, the evidence has suggested that mirtazapine may have efficacy in improving anxiety but primarily as an adjunctive agent.

Bupropion is a dopamine norepinephrine reuptake inhibitor approved for the treatment of MDD, attention-deficit/hyperactivity disorder (ADHD), and smoking cessation (31). Although bupropion has been used in patients with anxiety who are being treated with SSRIs as an adjunct to offset sexual side effects, there has been limited investigation of this medication as a monotherapy for anxiety. Although there is a common perception that bupropion can worsen anxiety, this may not be entirely accurate based on previous research of bupropion on anxiety symptoms in MDD when compared to SSRIs (31–33). To date, there is only one controlled trial of bupropion in anxiety disorders, a RCT comparing bupropion XL to escitalopram in GAD, which found that the two drugs had comparable anxiolytic efficacy (34). The evidence regarding efficacy of bupropion for the treatment of PD is conflicting (35, 36). Further work is needed to determine if bupropion and similar dopamine-enhancing agents are efficacious for the treatment of anxiety disorders.

Nefazodone, a serotoninergic modulating antidepressant thought to inhibit 5-HT reuptake and block postsynaptic 5-HT_2_ receptor (37), is only FDA-approved for the treatment of MDD. There have been several open-label studies suggesting potential benefit in PD and GAD but no controlled studies have been conducted (37–40). One RCT of nefazodone in SAD did not report separation from placebo (41). Overall, its use has been limited by concerns related to very rare but severe cases of liver toxicity.

### Gamma Aminobutyric Acid (GABA)

Benzodiazepines have been a longstanding treatment for anxiety and are still among the most widely prescribed class of psychiatric medications in the world (42, 43) although there has been increasing stigma surrounding the use of benzodiazepines in clinical practice (44). Critics of benzodiazepines cite their being prescribed as first-line treatments for anxiety in primary care settings before SSRIs, potential risks of tolerance, dependence, abuse or misuse, and concerns about falls in the elderly (45). However, there is a lack of strong evidence that SSRIs and other first-line treatments are superior to, or better-tolerated than, benzodiazepines for anxiety disorders, in particular GAD (46), especially for short-term treatment (44, 47), and possibly beyond 8 weeks as well (48). Benzodiazepines, which act as GABA-A agonists, are highly versatile medications that can be prescribed for a wide range of conditions including alcohol withdrawal, agitation or aggression, anesthesia, catatonia, mania, insomnia, muscle spasms, epilepsy or seizures, and REM sleep behavior and movement disorders (44). Although some reports suggest a potential risk of dementia associated with the chronic use of benzodiazepines, these have been called into question and it appears there is not an increased risk of neurocognitive disorders (49). Benzodiazepines are no longer considered first-line monotherapy for PD or other anxiety disorders but can be used in the short-term on either a standing or as-needed basis for PD, GAD, and SAD in conjunction with SSRIs and SNRIs (Table 1) (14–16). Caution is needed in children, geriatric patients, those with medical comorbidities, and individuals with substance use disorders, especially those using other central nervous system depressants like opioids and/or alcohol. Additionally, chronic use of benzodiazepines to treat anxiety with comorbid depression may result in reduced efficacy of antidepressants (50).

Anticonvulsants, some of which have GABAergic properties, include medications like pregabalin, gabapentin, tiagabine, lamotrigine, and topiramate. There is scant research on the use of this class of medications for anxiety disorders (51), with the strongest evidence for the use of pregabalin in GAD, including a meta-analysis of multiple RCTs reporting superiority to placebo and comparable effects to benzodiazepines (52). Pregabalin is thought to have anti-epileptic effects by its activity on the alpha-2 delta subunit of calcium channels to reduce neurotransmitter release (51). Pregabalin has FDA approvals for neuropathic pain, post-herpetic neuralgia, fibromyalgia, and as an adjunctive treatment for partial seizures. It was approved for GAD by the European Union in 2006, although since it was not approved by the FDA in the United States in 2009 the FDA application was withdrawn in 2010. Pregabalin was also shown to have potential efficacy in SAD (53), but only at doses of 450 or 600 mg, based on three randomized, double-blind, placebo-controlled trials (54–56). Pregabalin is generally well-tolerated, with the most common adverse effects being sedation, dizziness, and weight gain. Since there is the potential for abuse and dependence with pregabalin, it is listed as a Schedule V medication by the United States Drug Enforcement Administration (DEA), and providers need to be mindful of tapering the medication to prevent withdrawal, and to monitor prescribing in patients with substance use problems, especially opioids, for which there is an increased risk of overdose death (57–59).

Gabapentin, much like pregabalin, acts to modulate neurotransmitter release on voltage-dependent calcium channels (51). It is FDA-approved for the treatment of neuropathic pain, post-herpetic neuralgia, and partial seizures, but has been widely used off-label for various indications including fibromyalgia. There is increasing evidence for its use in alcohol use disorder, primarily for the treatment of withdrawal (60). Gabapentin has also been prescribed off-label for anxiety despite a lack of research evidence supporting such use (61). It was found to be efficacious in a small (*N* = 69) randomized, double-blind, placebo-controlled study in SAD (62). Another RCT of patients with PD found a difference between gabapentin and placebo but only in patients with severe panic symptoms (62). A third study found that gabapentin may help with anxiety related to public speaking (63). There are also several trials of gabapentin showing efficacy in perioperative anxiety (61). Gabapentin has similar adverse effects as pregabalin including sedation, dry mouth, constipation, weight gain, and pedal edema. Although gabapentin is not listed as a DEA controlled substance in the US, it may vary on how it is scheduled between states, and there is, like pregabalin, a risk of withdrawal and abuse potential, meaning caution must be used when prescribing this medication to patients taking opioids or those with substance use disorders (57, 59, 64). There are currently no known ongoing trials of gabapentin for anxiety disorders.

Another anticonvulsant, tiagabine, is FDA-approved for the treatment of partial seizures and has been shown to have potential anxiolytic effects in preclinical studies (65). Its mechanism of action is unknown, although it is thought to increase GABA activity by inhibiting GABA uptake in presynaptic neurons (51). While there have been promising open-label studies for PD, GAD, and PTSD (51), several RCTs do not support the efficacy of tiagabine in GAD (66) or PD (67). A small, randomized, double-blind crossover study with gabapentin and tiagabine in SAD reported that both drugs may be effective in reducing anxiety scores (68). There are no known active studies of tiagabine in anxiety disorders.

Lamotrigine is an anticonvulsant thought to inhibit voltage-dependent sodium channels causing decreased glutamate release (51). It is FDA-approved for the maintenance treatment of bipolar I disorder and for several types of seizure disorders. Lamotrigine was shown to have anxiolytic properties in preclinical studies (69). There are, however, very few studies of lamotrigine in anxiety disorders. One small case series reported improvement in symptoms in PD with agoraphobia (70). To date, no studies of lamotrigine for anxiety disorders are underway. Other anticonvulsants, like topiramate (thought to inhibit voltage-dependent calcium sodium channels to enhance GABA, block glutamate and inhibit carbonic anhydrase) have very limited data (51), mostly in open-label trials for SAD (71), although there is a trial listed on the Clinical Trials database for topiramate augmentation in treatment-refractory SAD (NCT00182455). There is only one known RCT of valproate, also known as valproic acid (which blocks voltage-dependent sodium channels and is thought to increase GABA by inhibiting glutamate-mediated excitation), in which is primarily used to treat bipolar disorder (51). In terms of GAD (72), valproic acid showed separation from placebo, and an open-label trial in SAD suggesting potential efficacy (73). Levetiracetam, approved only for seizure disorders (and with unclear mechanism, but thought to reduce hyperexcitation of brain cells through binding to the synaptic vesicle protein SV2A, but to not interfere with normal electrical activity) (51), has undergone two RCTs for SAD in which the medication failed to show a statistically significant difference compared to placebo (74, 75). There are no known RCTs of carbamazepine or oxcarbazepine in anxiety disorders.

### Antihistamines

Hydroxyzine is the most studied antihistamine for anxiety and the only antihistamine which is FDA-approved for use in anxiety. Antihistamines like hydroxyzine are histamine-1 receptor (H_1_) blockers that are commonly used as alternatives to benzodiazepines for anxiety, panic attacks, and insomnia, in both inpatient and outpatient settings (76). Hydroxyzine and other antihistamines like diphenhydramine may also be safer to use in children and adolescents and in pregnant women. There is, however, concern about the risk of anticholinergic toxicity or delirium in the elderly or patients with neurocognitive disorders. Antihistamines are generally well-tolerated, aside from adverse effects like dry mouth, constipation, sedation, and risks of use while driving. The primary drawback to this medication class is that patients tend to develop tolerance to antihistamines over time. A Cochrane review of 39 studies of GAD reported that hydroxyzine was superior to placebo and comparable to benzodiazepines and buspirone but the authors cited a high risk of study bias and concerns about sedation with hydroxyzine (76). To date, there have not been RCTs of hydroxyzine done in SAD and PD.

### Alpha- and Beta-Adrenergic Agents

Propranolol is a beta-adrenergic antagonist that is FDA-approved for multiple indications including hypertension, angina, atrial fibrillation and arrhythmias, migraine prophylaxis, and essential tremor (77). Although it is not approved for any psychiatric indications, it has been widely prescribed for SAD and performance anxiety. There is, however, a lack of research support for the use of propranolol in anxiety disorders (77) although it may still have a role in certain task-specific anxieties (78). Evidence for the use of other beta-blockers like pindolol in anxiety is sparse.

Clonidine and guanfacine are alpha-2 adrenergic receptor agonists FDA-approved for the treatment of hypertension (79, 80). Extended-release formulations of guanfacine and clonidine are also approved for ADHD in children and adolescents and clonidine is also approved for adjunctive use of cancer pain and is used off-label for management of opioid withdrawal. Clonidine has been studied previously as a research drug on the noradrenergic system in GAD and PD but was not found to have much utility in clinical settings (79, 81, 82). There have been no further RCTs of clonidine in anxiety disorders other than studies of the drug as pre-medication for children for preoperative anxiety (83). Both clonidine and guanfacine may have limited practical use in anxious patients given the lack of proven efficacy and concerns about hypotension and sedation.

### Antipsychotics

There is currently only one antipsychotic, trifluoperazine, a first-generation antipsychotic (FGA), which is FDA-approved for the treatment of anxiety. In spite of this, antipsychotics, most of which are dopamine-2 (D_2_) receptor antagonists, have been utilized on an off-label basis for multiple indications other than psychosis including anxiety (84). Several systematic reviews of antipsychotics in anxiety have reported that the majority of studies were of quetiapine, a second-generation antipsychotic (also with antagonism of the 5-HT_2_ and H_1_ receptors), in GAD, and showed the potential utility of quetiapine monotherapy in GAD despite poor tolerability (85, 86). The Canadian guidelines for anxiety and related disorders recommend olanzapine (D_2_, 5-HT_2_ and H_1_ antagonist), aripiprazole (a partial D_2_ and 5-HT_1A_ agonist and 5HT_2A_ antagonist) and risperidone (D_2_, and 5-HT_2_ antagonist), as augmentation strategies for GAD and PD (87). Risperidone and aripiprazole are also recommended as adjunctive drugs in the treatment of SAD (87). There is reasonable concern about the short- and long-term risks of using antipsychotics in anxiety disorders. First, there are limited studies to date in other anxiety disorders such as SAD and PD (85, 88). Second, it is unclear whether patients receive appropriate psychoeducation about the risks of tardive dyskinesia, extrapyramidal symptoms, neuroleptic malignant syndrome, weight gain, and metabolic syndrome. Further large-scale research and longitudinal studies of antipsychotics in anxiety are needed before these medications can be recommended.

## Novel Treatments for Anxiety Disorders

The focus of research on the pharmacotherapy of anxiety disorders has shifted from serotonin, norepinephrine and GABA systems to other neurotransmitters and pathways including glutamate and neuropeptides (89). Presented below is a review of recent and ongoing studies of medications for GAD, PD, and SAD. A summary of the findings is on Table 2.

**Table 2:** Novel medication treatments for anxiety disorders.

**Medication Class**	**Mechanism of action**	**FDA approvals**	**Past RCTs in anxiety**	**Ongoing/future trials in anxiety**
**Serotonergic agents:**				
Vilazodone	Selective 5-HT reuptake inhibitor, 5-HT_1A_ partial agonist (90)	MDD	GAD (90, 91) SAD (93) Sep. Anxiety (92)	SAD (NCT01712321)
Vortioxetine	Selective 5-HT reuptake inhibitor 5-HT_3_ antagonist 5-HT_1A_ agonist (95)	MDD	GAD (95–98) PD (99)	Comorbid SAD, MDD (NCT04220996)
Gepirone ER	5-HT_1A_ partial agonist (102)	None	None	GAD (103)
Tandospirone	5-HT_1A_ partial agonist (104)	None	None	GAD (NCT01614041)
PRX-00023	5-HT_1A_ partial agonist (106)	None	None	None
TGFK08AA	5-HT_1A_ partial agonist (89)	None	None	GAD (89)
TGW00AA	5-HT_1A_ partial agonist (107)	None	None	GAD, SAD (107)
AVN-101	5-HT_6_ receptor antagonist (108)	None	None	Anxiety Disorders (109)
Ondansetron	5-HT_3_ receptor antagonist (110)	Nausea/vomiting	GAD (110) PD (111)	None
Agomelatine	Melatonin-1/2 agonist, 5-HT_2C_ antagonist (112)	None	GAD (113)	None
Psilocybin	5-HT_2A_, 5-H_1A_, 5-HT_2C_ agonist (118)	None	“Life-threatening anxiety” (118)	Cancer-related anxiety (NCT00957359)
Lysergic diethylamide (LSD)	Unclear, modulates multiple 5-HT receptors (119)	None	None	“Life threatening anxiety” (NCT03153579)
**Glutamate:**				
LY354740	mGluR2-3 agonist (121)	None	PD (121)	None
LY544344	mGluR2-3 agonist (122)	None	GAD (122)	None
JNJ40411813 (ADX- 71149)	mGluR2 (+) allosteric modulator (123)	None	Anxious depression (123)	None
Ketamine	NMDA receptor antagonist (124)	MDD	SAD (137)	None
Riluzole	Inhibits glutamate release (143)	Amyotrophic lateral sclerosis	GAD (143–145)	None
Troriluzole (BHV-4157)	Reduces synaptic glutamate (NCT03829241)	None	GAD (NCT03829241)	None
D-cycloserine (DCS)	NMDA partial agonist (148)	Tuberculosis	PD, SAD and specific phobias (153–167)	None
Memantine	NMDA receptor antagonist (169)	Alzheimer's dementia	GAD (169)	None
Nitrous Oxide (N_2_O)	NMDA receptor antagonist (170)	Inhaled anesthetic	None	None
**GABAergic medications:**				
AZD7325	GABA-A alpha-2-3 modulator (NCT00808249)	None	GAD (NCT00808249)	None
PF-06372865	GABA-A (+) allosteric modulator (176)	None	GAD (176)	None
BNC-210	α_7_ nicotinic Ach (–) allosteric modulator, GABA modulator (177)	None	GAD (177)	None
SAGE-17	GABA-A (+) allosteric modulator (180)	None	None	GAD (179)
**Neuropeptides:**				
Oxytocin	Unclear	Labor induction	SP (184) SAD (188)	Anxiety + depression (NCT03566069)
LY686017	Neurokinin-1 antagonist (194)	None	SAD (194)	None
L-759274	Neurokinin-1 antagonist (195)	None	GAD (195)	None
Neuropeptide Y	Y1 agonist (197)	None	PTSD (199)	None
SSR149415	V1b antagonist (202)	None	MDD + GAD (202)	None
SRX246	V1a antagonist (203)	None	None	Experimental anxiety (NCT02922166)
Pexacerfont (BMS-562086)	CRF-1 antagonist (210)	None	GAD (210)	None
Verucerfont (GSK561679)	CRF-1 antagonist (NCT00555139)	None	GAD (NCT00555139)	None
Emicerfont (GW876008)	CRF-1 antagonist (NCT00555139)	None	GAD (NCT00555139)	None
Suvorexant	Orexin 1,2 antagonist (216)	Primary Insomnia	PD (NCT02593682)	None
**Neurosteroids:**				
Mifepristone (RU486)	Progesterone inhibitor (220)	Early pregnancy termination	PTSD, GAD, PD or anxiety NOS (220)	None
PH94B	Binds to nasal chemosensory receptors to trigger neural circuits (221)	None	SAD (221, 222)	Adjustment disorder with anxiety symptoms (NCT04404192)
**Cannabinoids:**				
Cannabidiol (CBD)	CB1 (–) allosteric modulator, CB2 antagonist-inverse agonist, 5HT_1A_ agonist (229)	None	SAD (26, 225, 227)	PD, GAD, SAD agoraphobia (NCT03549819)
Delta-9-tetrahydrocannabinol	CB1, CB2 partial agonist (229)	None	None	None
Dronabinol	CB1 agonist (229)	Chemo-related nausea/vomiting	None	None
Nabilone	CB1, CB2 agonist (229)	Chemo-related nausea/vomiting	GAD, “anxiety neuroses” (237, 238)	None
**Natural remedies:**				
Kava	Unclear, activity on Na, Ca channels or GABA-A receptor (244)	None	GAD (247)	None
Galphimine-B (G-B)	Unclear, inhibition of DA neurons in ventral tegmental area (250)	None	GAD (250)	Anxiety (NCT03702803)
Chamomile	Unclear, modulates GABA receptors (251)	None	GAD (251)	None
Lavender	Inhibition of voltage-gated Ca channels (249)	None	GAD (248, 249)	Dental anxiety (NCT04285385) Pre-operative anxiety (NCT03445130)
Saffron	Unclear, inhibiting 5-HT reuptake in synapses (252)	None	Anxiety symptoms (252)	GAD (NCT02800733)

*Key: 5-HT, Serotonin; Ach, Acetylcholine; CB, Cannabinoid Receptor; CRF, Corticotropin Releasing Factor; ER, XR, Extended Release; FDA, Food and Drug Administration; GABA, Gamma Aminobutyric Acid; GAD, Generalized Anxiety Disorder; MDD, Major Depressive Disorder; NMDA, n-methyl-d-aspartate; NRI, Norepinephrine Reuptake Inhibitor; NOS, Not Otherwise Specified; PD, Panic Disorder; RCT, Randomized Controlled Trial; SAD, Social Anxiety Disorder; Sep. Anxiety, Separation Anxiety Disorder; SNRI, Serotonin Norepinephrine Reuptake Inhibitor; SSRI, Selective Serotonin Reuptake Inhibitor*.

### Serotonergic Agents

The serotonin [5-hydroxytryptamine (5-HT)] system has been studied extensively in the etiology of anxiety and anxiety disorders, and the primary first-line pharmacotherapeutic agents for anxiety are serotonergic, including SSRIs, SNRIs, and azapirones like buspirone. There has been work on developing agents that work on several 5-HT receptors and may mimic the effects of SSRIs but with more favorable side effect profiles. For example, vilazodone, listed as an SSRI while also having partial agonistic properties on 5-HT_1A_, was approved by the FDA for the treatment of MDD in 2011 and it also has been studied in GAD and SAD (90). A meta-analysis of vilazodone for GAD reported that three 10-week RCTs that found a separation from placebo (90) but a later meta-analysis did not support the use of vilazodone in GAD (91). There was also a small pilot randomized, placebo-controlled trial of vilazodone in adult separation anxiety disorder which did not show significant separation between drug and placebo at 12 weeks but reported some differences in other anxiety measures (92). There has been one randomized, double-blind, placebo-controlled trial of vilazodone in SAD which showed potential efficacy and promise (93) and another study listed as active but not recruiting (NCT01712321). There are no known studies, active or recruiting, of vilazodone in PD. Vilazodone may confer benefits over SSRIs, including a possible lessened risk of sexual dysfunction (94), although gastrointestinal side effects may limit tolerability. Further comparison studies in anxiety disorders are needed.

Vortioxetine, a 5-HT_3_ antagonist and 5-HT_1A_ agonist, was FDA-approved for MDD in 2013, but its efficacy for anxiety is not clear (95). Despite initial promise in GAD (96, 97), and an initial meta-analysis recommending further study (95), two subsequent meta-analyses failed to show significant efficacy of vortioxetine over placebo in GAD despite fairly good tolerability (91, 98) and thus further study and pursuit of FDA approval in GAD was abandoned in 2015. There was one open-label study of vortioxetine in PD reporting improvement in panic symptoms (99) but no known RCTs. There is one study in SAD comorbid with MDD underway (NCT04220996), but no studies of vortioxetine in other anxiety disorders.

As noted above, buspirone has been FDA-approved for the treatment of anxiety, which led to the consideration of other azapirones (100). Gepirone, an azapirone and a selective 5-HT_1A_ receptor partial agonist, in its extended-release (ER) form, has been studied previously as an antidepressant, although it has been shown to have also anxiolytic properties (101, 102). Gepirone ER is currently in Phase 2 trials for GAD, MDD, and hypoactive sexual desire disorder (103). Tandospirone, yet another azapirone studied for depression and anxiety, showed promise in reducing anxiety in patients with anxious depression (104, 105). There was one completed trial of tandospirone in GAD but no published results yet (NCT01614041). There are also no recent or active, ongoing studies of other azapirones such as ipsapirone or lesopitron. In terms of non-azapirone 5-HT_1A_ agonists, PRX-00023 was studied in GAD in a RCT and, despite good tolerability, it did not show separation from placebo on endpoint anxiety (106). Additionally, there are other selective 5-HT_1A_ partial agonists, such as TGFK08AA, in development for GAD (89), and TGW00AA (FKW00GA) in Phase 2 studies for GAD and SAD (107).

Some 5-HT_6_ receptor antagonists, such as AVN-101 and AVN-397, have been reported to have anxiolytic properties in animal studies (108). AVN-101 was shown to be safe and well-tolerated in Phase 1 studies and is under investigation for Alzheimer's Disease and may have potential for study in anxiety disorders due to its anxiolytic effects (109).

Ondansetron, approved to treat nausea and vomiting, is a selective 5-HT_3_ antagonist that was found to improve anxiety in a small RCT in GAD (110) and an open-label trial in PD (111). There have, however, been no further studies of ondansetron in anxiety disorders, with the only recent and current studies focused on its use in OCD and tic-related disorders.

Agomelatine, a melatonin-1/melatonin-2 agonist and 5-HT_2C_ receptor antagonist, has been studied more extensively in depression, although it was also found to have anxiolytic properties (112). Based on several RCTs including comparisons with escitalopram (113–117), a meta-analysis of treatments for GAD determined that agomelatine was well-tolerated and potentially efficacious with the caveat of small sample sizes (91). Despite its apparent positive response in GAD, there are no known controlled studies of agomelatine in PD or SAD or any known ongoing trials in anxiety disorders.

Finally, it is worthwhile to discuss hallucinogens, which act on serotonin receptors, and their anxiolytic potential. Psilocybin (4-phosphoryloxy-N,N-DMT) is an indolealkylamine derived from mushrooms that causes perceptual changes in humans (altered thinking, synesthesia, illusions) and is listed as a DEA Schedule I controlled substance. It has been shown to have the potential to reduce anxiety, with several completed RCTs of psilocybin in cases of “life-threatening anxiety” (patients with anxiety who have life-threatening diseases like cancer) (118). Lysergic acid diethylamide (LSD) is a widely abused hallucinogen with similar effects as psilocybin and is also a Schedule I drug. Prior evidence for the use of LSD in anxiety is less robust and favors its use in alcohol use disorder (119). There is an active clinical trial of psilocybin in cancer-related anxiety (NCT00957359) and a randomized, double-blind, placebo-controlled trial testing LSD in patients with anxiety with or without life-threatening diseases (NCT03153579). The difficulties regulating Schedule I drugs may limit whether hallucinogens have potential for widespread clinical application.

### Glutamate Modulators

Glutamate is the primary excitatory neurotransmitter of the central nervous system. Receptors for glutamate include ionotropic receptors [N-methyl-D-aspartate (NMDA), α-Amino-3-hydroxy-5-methyl-4-isoxazolepropionic acid (AMPA)/kainate and metabotropic receptors (mGluR)]. Several preclinical studies have reported anxiolytic effects of mGluR modulators (120), although human studies have not been as promising. For example, LY354740, an mGluR 2-3 agonist, did not separate from placebo in a randomized controlled comparison study with paroxetine (121) in patients with PD. After studies with LY354740 were halted due to concerns about bioavailability, LY544344, a pro-drug of LY354740, was studied for GAD in an 8-week randomized placebo-controlled trial with reported efficacy but the study was halted due to concerns about convulsive activity in preclinical trials (122). JNJ-40411813 (ADX-71149), an mGluR2 positive allosteric modulator, was studied in a RCT for anxious depression but did not show efficacy (123).

Ketamine, originally developed as an anesthetic, has shown rapid and robust antidepressant effects in multiple randomized controlled clinical trials. The majority of these studies have tested the safety and efficacy of a single intravenous (IV) infusion of the ketamine in adults with treatment-resistant depression (TRD), most commonly at a dose of 0.5 mg/kg (124–127). Subsequent studies provide evidence for a favorable safety and efficacy profile of repeated doses of ketamine administered over several weeks (128–130). It should be noted that the use of IV ketamine for TRD is off-label and there is currently a lack of data concerning the longer-term safety and efficacy of this approach (124, 131). Ketamine exists as a 1:1 racemic mixture of R-ketamine and S-ketamine. In 2019, an intranasal (IN) form of S-ketamine (“esketamine”) gained approval from the FDA for the treatment of MDD in adults with treatment-resistant depression (132). Buoyed by these observations in depression, recent studies have examined the tolerability and potential efficacy of ketamine in anxiety disorders, based on preclinical observations (133).

An early study reported the benefit of daily oral ketamine on symptoms of both depression and anxiety in adults in hospice care (134). A small open-label study suggested benefit of ketamine administered subcutaneously in a single ascending dose design in patients with refractory SAD and/or GAD (135). The same group showed preliminary benefit of ketamine 1 mg/kg injected subcutaneously dosed once or twice weekly for 3 months among patients who had responded in the initial ascending dose study (136). More recently, a double-blind RCT of intravenously administered ketamine at 0.5 mg/kg compared to saline placebo showed benefit in patients with SAD measured using the Liebowitz Social Anxiety Scale (LSAS) (137). There are no known ongoing trials of ketamine in PD, GAD, or SAD.

Riluzole, a glutamate modulator approved for the treatment of amyotrophic lateral sclerosis (ALS), also has been studied previously as an adjunctive agent in TRD (138–140). Animal studies supported the efficacy of riluzole in models of anxiety (141, 142). To date, there is only one trial of riluzole in anxiety disorders which was an open-label study of 18 participants with GAD. The trial reported response or remission in a majority of patients, as measured on the Hamilton Anxiety Scale, although the results are limited by the lack of a control group and the small number of participants (143). Subsequent functional neuroimaging studies of open-label riluzole in GAD reported that patients experienced changes in hippocampal volumes and N-acetylaspartate (NAA) concentrations which correlated with improvement on anxiety scales compared with healthy volunteers (144, 145). An analog of riluzole, troriluzole (BHV-4157), underwent a Phase III trial in GAD which has been completed although the results have not been published (NCT03829241).

There are several animal studies of AMPA modulators, including PEPA, primarily in fear extinction models of anxiety, showing positive anxiolytic effects (146). One AMPA modulator, Org 26576, has been studied for MDD (147). To date, however, there are no known studies in the pipeline for AMPA modulators for anxiety disorders.

D-cycloserine (DCS), an NMDA partial agonist, is among the most widely studied glutamatergic agents in anxiety (148). D-cycloserine is unique in that research to date has focused on the effects of DCS on anxiety in the context of psychotherapy or fear learning. In animal and human studies, DCS has been shown to facilitate fear extinction (148). Although not known to be efficacious as a monotherapy (149), DCS at a low dose of 50 mg/day (which is largely an NMDA agonist) has been successfully used in augmentation of exposure therapy or CBT, including reducing anxiety in persons performing cognitive tasks (150) and facilitating declarative learning (151) by reducing reactivity to phobic stimuli in persons with specific phobia on functional MRI (152). D-cycloserine has been studied for augmentation of psychotherapy in PD, SAD, and specific phobias. A 2015 Cochrane review reported no difference between DCS and placebo in augmentation of cognitive and behavioral therapies in anxiety and related disorders at study endpoint or follow up, in both children and adolescents (153). A 2017 meta-analysis of DCS in anxiety disorders found a small difference between DCS and placebo post-treatment but minimal gains on follow-up treatments (154). Subsequent studies of DCS augmentation in PD have been mixed (155, 156). In SAD, despite earlier studies showing promise for the use of DCS augmentation of exposure therapy (157–159), there have been far less encouraging findings in subsequent studies (157, 160–162). Studies of DCS in SP, including acrophobia and spider phobia, have yielded inconsistent but mostly negative findings for DCS compared to placebo (163–167). There are no active clinical trials of DCS augmentation in PD, GAD, SAD, or SP. On balance, while initial studies of DCS showed promise for use in augmentation of psychotherapies, subsequent larger-scale studies have been disappointing.

Memantine, an NMDA receptor antagonist FDA-approved for the treatment of Alzheimer dementia, moderate-severe, was tested previously in preclinical studies as a potential antidepressant (168). One study reported minimal improvement in seven patients with GAD, while 10 participants with OCD experienced modest benefit (169). There are no known active studies of memantine for anxiety disorders.

Finally, nitrous oxide (N_2_O), an inhaled anesthetic most often used in dental procedures, is a NMDA receptor antagonist (170). Nitrous oxide can be used recreationally and has been associated with potential neurologic and psychiatric adverse effects (171). There also has been some study of N_2_O for the treatment of alcohol withdrawal (172) and a proof-of-concept study in MDD (173). Although there is literature on using N_2_O for the treatment of dental phobia and other procedure-related anxiety (174), there is, to date, no known past or current study of N_2_O as a pharmacologic treatment of PD, GAD, or SAD.

### GABAergic Medications

Given how efficacious benzodiazepines (GABA-A agonists) are for the treatment of anxiety disorders, and how there may be potential benefits with pregabalin and gabapentin, there has been an effort to find novel GABAergic anxiolytic agents. To date, however, several GABA-A receptor subtype agonists have either failed to reach market due to lack of efficacy or poor tolerability (89, 175). AZD7325, a GABA-A alpha-2-3 modulator, failed to separate from placebo in a Phase 2 comparative trial with placebo and alprazolam for GAD (NCT00808249). PF-06372865, a GABA-A positive allosteric modulator, tested at two doses, failed to separate from placebo as an adjunctive treatment in patients with GAD (176). On an encouraging note, BNC-210 (IW-2143), an α7 nicotinic acetylcholine receptor–negative allosteric modulator which also modulates the GABA receptor, was reported to result in reduced amygdalar activation to fearful faces compared to placebo and comparably to lorazepam, in patients with GAD (177). Although it is unknown how many GABA modulators are being studied in anxiety, preclinical research suggests that several agents may be in the pipeline (178). For instance, SAGE-217 is a GABA-A positive allosteric modulator that is under Phase III study for MDD and postpartum depression and is being explored for treatment of GAD (179, 180). Finally, phytochemical (herbal) compounds that have GABAergic properties are also under investigation (see “Natural Remedies” section below).

### Neuropeptides

Neuropeptides are small proteins that work as neuronal signaling molecules and are involved in an array of brain functions such as analgesia, reward systems, social behaviors, learning, and memory. In addition, specific neuropeptides such as oxytocin, substance P, neuropeptide Y (NPY), arginine vasopressin (AVP), and cholecystokinin (CCK) play significant roles in modulating fear and anxiety.

Oxytocin is a neuropeptide involved in attachment and pro-social behaviors. Due to its poor absorption in the digestive tract, oxytocin must be administered either intravenously, intranasally or sublingually, where it is well-tolerated with no known serious adverse effects (181). Studies have shown that in healthy adults, oxytocin has positive effects on emotion modulation (182), and that low oxytocin has been associated with high anxiety (183). Animal studies suggest that oxytocin has anxiolytic effects and human research suggests that oxytocin may increase anxiety acutely. For example, a double-blind, placebo-controlled study of intranasal oxytocin on single-session exposure-based psychotherapy for arachnophobia found that oxytocin impaired treatment response compared to placebo (184). Overall, however, oxytocin may have overall positive effects on anxiety depending on the frequency and context of administration (183). Research on oxytocin for the treatment of anxiety disorders has been focused on SAD (185, 186), with studies reporting increasing amygdalar-prefrontal activity in response to emotional faces (187), and enhancing prosocial behaviors (188). Although there is a large body of research on the use of oxytocin for augmentation of antipsychotics in schizophrenia (189), several of these trials have been called into question (190) due to study design and sample size and there has been controversy about whether oxytocin can be absorbed into the brain with intranasal administration or if peripheral levels reflect central activity (191). Currently, there is one known study evaluating intranasal oxytocin in patients suffering from acute anxiety and depression during psychiatric hospitalization (NCT03566069).

Substance P is one of the major neuropeptides found in the nervous system (192). Given its abundance in the fear center of the brain, Substance P and its neurokinin receptor system have been a great topic of interest in anxiety disorder research (193). Despite ample research interest, several trials have failed to demonstrate the efficacy of Substance P in reducing symptoms of anxiety disorders (194, 195), which has led to a decrease in pharmaceutical studies (193). A recent study conducted by Frick et al. (196) however, found that individuals with SAD demonstrated more NK1 receptor availability in the right amygdala when compared to healthy controls. This finding highlights the need for further research on utilizing NK1 receptor antagonists for the treatment of some anxiety disorders. Currently, there are not any ongoing clinical studies that evaluate the use of Substance P for the treatment of anxiety disorders.

Neuropeptide Y is one of the most abundant neuropeptides in the brain and numerous reports have found that NPY is critical for the stress adaptation process (197). In humans, NPY has been closely linked to trauma. Most recently, a small RCT of intranasally administered NPY in patients with MDD showed potential benefit for depression (198). A study conducted by Sayed et al. found that intranasally administered NPY was associated with greater treatment response and improvement in anxiety ratings when compared to placebo in individuals with PTSD (199). This finding encourages future research that studies the safety and efficacy of NPY as an anxiolytic treatment. There are however no active trials of NPY in GAD, PD, or SAD.

Arginine Vasopressin (AVP) has been shown in animal models to be related to anxiety responses and vasopressin V1a and V1b receptor antagonists may have anxiolytic properties (200, 201). Few human studies of V1 antagonists have been published. Griebel et al. (202) compared the vasopressin V1b antagonist, SSR149415, in a randomized, double-blind study, to escitalopram, paroxetine and placebo, in MDD and GAD, reporting that SSR149415 did not differentiate from placebo in outcome measures for GAD. Conversely, studies conducted by Fabio et al. (203), and Lee et al. (204) have shown safety and promise of V1a receptor SRX246 as an anxiolytic, with an active study of this compound in an experimental model of anxiety in humans (NCT02922166). There are currently no known clinical trials studying V1a antagonists in the treatment of anxiety disorders.

Cholecystokinin (CCK) is a peptide which helps regulate gastric secretions and motility and biliary function in the gastrointestinal system; in the brain, CCK is found in the brain's fear network (e.g., the amygdala, hypothalamus, etc.) (205). Current research surrounding CCK is complex in that CCK agonists are panicogenic, but CCK-2 antagonists fail to alleviate human anxiety (206), leaving many unanswered questions regarding the role of CCK in human anxiety. According to clinialtrials.gov, there are currently no known clinical studies evaluating CCK antagonists for the treatment of anxiety disorders.

Corticotropin-releasing factor (CRF) plays a role in stress response and individuals with anxiety disorders exhibit aberrant CRF homeostasis (207). Conversely, CRF receptor antagonists have been shown to have potential anxiolytic effects in animal models (208). The results from preliminary research in anxiety disorders, however, have been disappointing (209). One randomized, double-blind trial of the CRF-1 antagonist, pexacerfont (BMS-562086), compared to escitalopram and placebo for the treatment of GAD, found that it did not separate from placebo (210). There are also completed, but not published, studies for SAD comparing CRF-1 antagonists verucerfont (GSK561679) and emicerfont (GW876008) to alprazolam and placebo (NCT00555139), and a Phase 2 trial of patients with GAD comparing GW876008 to paroxetine and placebo (NCT00397722). Research on CRF-1 antagonists appears to have shifted toward addiction with recent trials of pexacerfont and verucerfont in alcohol use disorder (211, 212). There are no known active trials of CRF-1 antagonists in anxiety disorders.

Orexin, also known as hypocretin, is a neuropeptide associated with arousal, appetite, and wakefulness (213). Orexin is also thought to play a role in stress response with alterations in orexin found in depression and anxiety (214). Based on the research finding that orexin levels are increased in CSF of individuals with PD (215), orexin is thought to have anxiogenic effects, leading to research on orexin-1 and orexin-2 receptor, and dual-receptor antagonists for treatment of anxiety disorders (216). Suvorexant, an orexin-1 and orexin-2 receptor antagonist, is FDA-approved for primary insomnia, and is under study in PD, comparing the drug to placebo to monitor orexin levels and response to a carbon dioxide challenge (NCT02593682). There are no other known ongoing studies of orexin antagonists in anxiety disorders.

In summary, neuropeptides appear to be a promising emerging field for the treatment of anxiety disorders but there no clear therapeutic candidates for anxiety disorder have been identified as of yet.

### Neurosteroids

Neurosteroids, also known as neuroactive steroids, act as transcription factors to regulate gene expression and endogenously modulate neuronal excitability by interacting with GABA-A, NMDA, and glutamate receptors (217). Both preclinical and clinical studies have demonstrated evidence of aberrant neurosteroid homeostasis in anxiety disorders (218). The antidepressant effects of SSRIs have been shown to correlate with increased brain and cerebrospinal fluid levels of allopregnanolone, a neurosteroid with potent modulatory activity on GABA-A receptors (219). Because of their ability to rapidly control the excitability of the central nervous system, there has been increasing interest in the role of neurosteroids as novel treatments for anxiety disorders. To date, two compounds with neurosteroidal activity, mifepristone (RU486) and PH94B, have been investigated for the treatment of anxiety symptoms.

RU486 is a progesterone inhibitor used for early pregnancy termination (220). It is a glucocorticoid antagonist and has been studied for MDD with psychotic features, improving cognition in patients with bipolar disorder and schizophrenia, and for the treatment of PTSD. A 12-week, pilot clinical trial assessed the effect of mifepristone in older adults as a treatment for anxiety disorders with co-morbid cognitive dysfunction (220). The study included 15 older adults (ages 60 years and older) with an anxiety disorder (GAD, PD, or anxiety disorder not otherwise specified). Subjects were randomized to either mifepristone 300 mg daily or placebo for the first week of the study. After the first week, all patients received treatment with mifepristone 300 mg daily for an additional 3 weeks. At the end of week 4, mifepristone was discontinued, and follow-up assessments of memory, executive function, and anxiety were completed at week 12. Subjects with higher baseline cortisol levels had improvements in memory, executive function, and anxiety, while those with low or normal baseline cortisol levels experienced little to no mifepristone-related improvement. There are no active studies of mifepristone in anxiety disorders.

PH94B, an intranasally administered neurosteroidal aerosol, has been investigated for the acute treatment of SAD. In a phase 2, multi-center, randomized, double-blind, placebo-controlled, single-dose study, 91 females (ages 19–60 years) with SAD were randomized to receive either placebo or PH94B nasal spray 15 min prior to a public speaking or social interaction challenge under laboratory settings (221). Seventy-five percent of the subjects who received PH94B were considered responders [either “much” or “very much improved” as measured by the Clinical Global Impressions-Improvement (CGI-I)], compared with 37% of the subjects who received placebo. There were no differences in adverse events reported by the PH94B and placebo groups. A second study assessed the use of PH94B for the treatment of symptoms of SAD in real-world settings in a pilot, randomized, double-blind, placebo-controlled trial (222). Twenty-two males and females (ages 18–65 years) with SAD were randomized to either placebo or PH94 nasal spray. The subjects were instructed to self-administer the nasal spray 15 min prior to a distressing social interaction or performance, up to four times per day. PH94B was superior to placebo in decreasing mean peak levels of symptoms of social anxiety, as measured by subject-reported subjective units of distress (SUDs). The results of an open-label study of PH94B for the treatment of adjustment disorder with anxiety symptoms in adults which has planned to begin enrollment in 2020 will be of great interest (NCT04404192).

### Alpha- and Beta-Adrenergic Agents

As noted above, clonidine may be used off-label for anxiety but there have been no recent clinical studies of clonidine in anxiety disorders. There are no current drug trials of clonidine in PD, SAD, GAD, or SP. One RCT of guanfacine ER in children and adolescents (ages 8–17 years) with GAD, separation anxiety disorder, and SAD reported that the medication was safe and well-tolerated but it is unclear whether it is efficacious (80). To date, there are also no known active studies of guanfacine in anxiety disorders. As noted earlier, there is currently limited active investigation of propranolol for anxiety disorders outside of the extensive research done on memory consolidation and PTSD.

### Cannabinoids

In many parts of the world, cannabis is consumed for its euphoric and relaxing effects. There is a widespread belief that cannabis, cannabidiol (CBD), and other cannabinoids are harmless substances and can lower anxiety and induce relaxation. However, the literature does not support the belief that cannabinoids are safe for patients with anxiety disorders, nor does it support the notion that cannabinoids improve anxiety and related symptoms in those patients. The quality of evidence currently available from clinical trials with cannabinoids and anxiety is very low. This is in part because most of the studies included participants with a primary diagnosis of chronic non-cancer pain, multiple sclerosis or fibromyalgia, rather than anxiety disorders. Other limitations of these studies include small sample size and other methodological flaws (223).

Endogenous and exogenous cannabinoids act on the cannabinoid type 1 (CB1) receptor, serotonergic type 1A (5HT_1A_) receptor and the transient receptor potential vanilloid type 1 (TRPV1) receptor. Cannabinoid type 1 receptor agonists have a biphasic effect: they have anxiolytic properties in low doses and anxiogenic properties in high doses. While the activation of the CB1 receptor produces inhibitory effect in the neuron, leading to an anxiolytic effect, high doses of CB1 receptor agonists induce activation of TRPV1 receptor, which produces anxiogenic effects (224). Several drugs that act as 5HT_1A_ receptor agonists proved to be effective in the treatment of anxiety disorders; recent studies indicate that cannabidiol and other cannabinoids also act on the 5HT_1A_ receptor, potentially resulting in anxiolytic effects (224).

The most studied cannabinoid in anxiety is CBD. Preclinical animal model studies and human trials indicate that CBD is a potentially effective treatment for PD, GAD, and SAD (224). In the study from Bergamaschi et al. (225), the authors found that the administration of a single dose of CBD (600 mg) successfully attenuated the anxiety response to a public speaking test in subjects with SAD. In another study, the same research team found that a single 400 mg dose of CBD reduced the anxiety associated with a SPECT scan in patients with SAD (226). One small RCT which included 37 adolescents with SAD (CBD *n* = 17, placebo *n* = 20) showed promising results (227). Subjects received 300 mg of CBD or placebo daily for 4 weeks. The Liebowitz Social Anxiety Scale (LSAS) scores decreased by 16% (*P* = 0.03) pre- and post-treatment in the CBD group. The drug-placebo difference in LSAS was not statistically significant. Currently, there is a RCT with CBD underway (NCT03549819) with an estimated completion date of October 2020. Flexibly dosed CBD (200–800 mg/day) will be administered for 8 weeks to patients with PD, GAD, SAD, or agoraphobia.

Excessive activity in limbic and paralimbic cortical areas has been consistently implicated in the pathophysiology of anxiety disorders. The parahippocampal gyrus and hippocampus are thought to play a key role in mediating fear and anxiety. The public speaking test produces activation of limbic areas in SAD subjects, but in subjects treated with citalopram there is a decreased regional cerebral blood flow (rCBF) response in the amygdala, hippocampus, and the periamygdaloid, rhinal and parahippocampal cortices (228). In the study from Crippa et al. (226) they found that CBD administration decreased resting rCBF in the limbic area, namely in the left parahippocampal gyrus and hippocampus. Although the effects of citalopram and CBD were similar in some areas, citalopram produced decreased rCBF in the cingulate gyrus, while CBD produced increased rCBF in the right posterior cingulate gyrus (226).

The anxiolytic effects observed with CBD are in contrast to the anxiogenic effects induced by delta-9-tetrahydrocannabinol (229). Delta-9-tetrahydrocannabinol (THC) is a partial agonist of the CB1 receptor that can have anxiolytic effect in low doses, but in high doses can induce anxiety and panic attacks (229). In a clinical trial for treatment of Tourette syndrome, the authors found worsening of obsessive-compulsive behavior after the administration of THC. No significant differences were found in anxiety scores, but there was a trend of higher phobic anxiety after administration of THC (230). There are no registered trials for THC in anxiety disorders.

Dronabinol is a synthetic trans isomer of THC and, compared to THC has higher affinity to the CB1 receptor (229). Two RCTs found that dronabinol was effective for treatment for pain, but neither study found significant changes in anxiety scores (231, 232). In the study by Narang et al. two subjects experienced high anxiety as a side effect of a higher dronabinol dose (20 mg) (232). There is one registered study listed as withdrawn (NCT03369639) with dronabinol and anxiety disorders; there are no active studies.

Nabilone is also a synthetic cannabinoid which is similar to but more potent than THC. It has high affinity to CB1 and CB2 receptors. Four studies assessed the effects of nabilone in pain as a primary outcome and anxiety as a secondary outcome (233–236). One RCT (233) which included subjects with neuropathic pain demonstrated that nabilone was effective for the treatment of pain but did not produce any changes in anxiety. In the study by Toth et al. 1–4 mg/day of nabilone was effective in relieving pain, improving disturbed sleep, reducing anxiety, and increasing the quality of life of patients with diabetic peripheral neuropathic pain (236). Skrabek et al. (235) also found that nabilone produced improvement of pain, decreased anxiety, and increased quality of life in fibromyalgia patients. In patients with chronic headache, nabilone was more effective than ibuprofen for pain management, but there were no significant differences in anxiety scores (234). In the first of two small clinical trials (237), patients with either GAD or “anxiety neuroses” received placebo or nabilone over four sessions, 7 days apart. Anxiolytic effects were observed only in a small portion of the patients. In another study, 25 patients with anxiety received 2–10 mg/day of nabilone for 28 days and had significant improvement of anxiety in an open-label phase (238). In the RCT phase of the same study, patients received nabilone 3 mg/day for 28 days and the improvement in the nabilone group was superior to the placebo group. Side effects included drowsiness, dry mouth, and dry eyes. Significant improvements in anxiety scores were also noted in a cross-over comparison of nabilone (1–2.5 mg twice daily) and placebo in 11 anxious patients (239). There are currently no registered clinical trials assessing nabilone in anxiety disorders.

Preparations including both THC and CBD (THC-CBD) have been tested as treatments for pain in two RCTs. In two small RCTs which included patients with multiple sclerosis, THC-CBD did not increase or decrease anxiety (240, 241). In one of those studies (240), THC-CBD was effective in the treatment of pain and sleep disturbances, while in the second study (241) THC-CBD was not effective for pain. In a RCT of THC-CBD for the treatment of Huntington's Disease (242), there were no significant improvements of motor, cognitive, behavioral, and functional scores. Also, there were no significant changes in anxiety scores. There are no registered trials for THC-CBD in anxiety disorders, but there is one trial underway (NCT03491384) assessing the effect of recreational cannabis use on anxiety symptoms.

Regarding the safety of cannabinoids, currently there are no studies showing that CBD or nabilone increase anxiety or cause panic attacks, suggesting that these medications are safe for patients with anxiety disorders. However, THC and dronabinol are likely not safe drugs for these patients because they can induce anxiety and panic attacks depending on the doses and individual predispositions of each patient. In conclusion, cannabidiol and nabilone are the most promising cannabinoids in the treatment of anxiety disorders, but the level of evidence for these drugs is still very low. Overall, THC, THC-CBD and dronabinol seem to be ineffective and potentially harmful for subjects with anxiety disorders.

### Natural Remedies

There has been increased study of herbal medications for depression and anxiety, with over 30 medications having been tested in some capacity over the past 15 year (243). Despite this burgeoning interest in natural agents, the data for most of them remains sparse. The most widely studied herbal compound is kava, a plant containing kavapyrones, which are thought to exert anxiolytic effects through activity on sodium and calcium channels or most likely from action on GABA-A receptors (like benzodiazepines) (244). A Cochrane meta-analysis of RCTs of kava in anxiety disorders, published in 2003, reported reductions in anxiety scores and separation from placebo (245). A more recent analysis was more conservative and noted that kava could be recommended for short-term use in anxiety but should not replace longer-term medications (246), while another review concluded that, given insufficient evidence, kava could not be recommended for GAD (247). All the above reviews also noted the risk of liver toxicity, including potentially severe liver toxicity, with the use of kava. There are no known active or upcoming studies of kava in anxiety disorders.

Several systematic reviews of other herbal compounds for the treatment of anxiety and anxiety disorders have been conducted and reported including RCTs for several agents such as ashwagandha, passionflower, galphimia, echinacea, ginkgo, chamomile, lemon balm, valerian, and lavender (243, 248, 249). A recent trial compared Galphimine-B (G-B), isolated from Galphimia (and found in rats to have anxiolytic effect through inhibition of dopaminergic neurons in the ventral tegmental area), to alprazolam, a benzodiazepine, as a control, in patients with GAD and reported that G-B had comparable efficacy to alprazolam with less sedation (250). Although there have been several positive studies of natural treatments for GAD, in particular for chamomile (251), the agent with strongest evidence for use is kava. Currently, there are ongoing clinical trials using lavender (thought to treat anxiety by inhibiting voltage-gated calcium channels) for dental (NCT04285385) and pre-operative anxiety (NCT03445130) and a Phase 2 randomized double-blind trial comparing galphimia to alprazolam for the treatment of anxiety (NCT03702803). Although saffron (*Crocus sativus*) has been studied for depression and anxiety, due to its possible effects of inhibiting serotonin reuptake in synapses (252), there is only one listed study, a randomized, double-blind RCT in mild to moderate GAD, although its status is unknown (NCT02800733).

## Discussion

Since this group's last review of novel therapies for anxiety disorders in 2014 (253), there has been little headway made in the development or clinical evaluation of new drugs for PD, GAD, and SAD. There have been no new medications approved by the FDA for any anxiety disorder during that time. Although there have been trials of serotonergic agents (like vortioxetine and agomelatine), glutamate modulators (such as riluzole and ketamine), neuropeptides, and even cannabinoids, very few have advanced to Phase III trials or have shown real promise for anxiety disorders. This trend lies in contrast to the greater number of studies taking place for PTSD, OCD, mood/depressive disorders, and schizophrenia. Moreover, the field lacks data contrasting specific drugs (or mechanisms of action) with efficacy, which would be required to propose rational protocols for the selection of optimally efficacious treatments.

The traditional areas of research for anxiety included serotonin, norepinephrine, and GABA, and indeed there are several drugs recently studied and under investigation targeting these neurotransmitters. This research, however, has been built upon the previous success of SSRIs, SNRIs, and GABAergic agents like benzodiazepines, and, to a lesser extent, pregabalin and gabapentin, neither of which are approved in the United States but are prescribed off-label for anxiety. The temptation to continue work on these pathways is due to the acknowledgment that these treatments are effective while failing to expand beyond this comfort zone. Indeed, the neurobiology of anxiety has expanded well beyond the research on fear condition, false suffocation alarms, and the neuroendocrine and HPA-axis models of panic and fear. The early neurocircuitry models of anxiety were based on pre-clinical research and cast a wide net, including PTSD in those models. It is now better understood how much heterogeneity exists between PTSD and other anxiety disorders and even in the class of anxiety disorders, among PD, SAD, and GAD (11). While neuroimaging studies in the last two decades have led to a refined understanding of brain circuits involved in fear and anxiety, this knowledge has not yet translated in insights leading to novel treatments (with the exception, perhaps, of attempts to use transcranial magnetic stimulation to modulate anxiety and fear circuitry) (254). This is also because in general pharmacotherapies are less clearly related to the functioning of specific brain circuits.

The pursuit of novel pharmacotherapies for anxiety disorders has been fraught with many complications. The first-line treatments, SSRIs and SNRIs, were originally approved for depressive disorders and then later for anxiety disorders. There have been very few drugs developed *de novo* for anxiety. Studies of newer agents have been hampered by flawed study designs such as lack of controls or using placebos instead of comparison to established medications such as SSRIs or benzodiazepines. While this may seem like an insurmountable hurdle, there is hope in that several compounds, including neurosteroids, neuropeptides, and phytochemicals (herbal compounds), have shown some potential. It also would help to study medications specifically for how the disorder manifests clinically, such as how PH94B has been investigated for performance anxiety in SAD by being administered 15 min before the participant is to have a social interaction or give a performance (222). Although study designs may be tricky, studying the use of pro re nata (PRN) or “as-needed” medications may be of more clinical use to patients, especially given that this is how a significant portion of patients are prescribed benzodiazepines.

Therein lies a second area of concern. Although it has been assumed that most patients respond to SSRI or SNRI medications, benzodiazepines, psychological treatments, or some combination, about one-third of these patients have treatment-refractory anxiety. There is still little known about treatment resistance in anxiety disorders and how to treat it effectively. It also remains unclear how many patients are being treated with effective doses or being given adequate trials or are potentially being misdiagnosed or treated with inappropriate regimens. Anxiety disorders, in addition to their high prevalence, are a leading cause of disability, which is exacerbated by their high comorbidity with depression (255). Naturalistic studies may be needed to understand how to treat anxiety patients with psychiatric, medical, and substance comorbidities.

Perhaps the clearest limitation of this synopsis is the intentional omission of psychotherapies for anxiety disorders. Although their efficacy in PD, GAD, and SAD, has been documented, this review aimed to focus on pharmacotherapies. That said, it is impossible to ignore the importance of therapy-assisted medications such as DCS or potentially even psychedelic medications. Ideally, such medications could reduce the distress related to exposure techniques and enhance the retention of information in anxiety-focused psychotherapy, ultimately increasing its overall efficacy. Such medications are also important given the lack of access to care and how few patients are in fact being treated first by psychiatrists (for medication management) and can receive appropriate CBT or exposure therapies for their anxiety disorders. This is certainly an area that needs greater investigation. Further research on augmented psychotherapies should not, however, preclude the concomitant development of novel pharmacotherapies, especially given evidence for greater efficacy of pharmacologic treatments over psychological therapies for certain anxiety disorders such as GAD (256).

Since this review did not uncover a wide range of support for promising pharmacotherapies for anxiety disorders in development, we need to reconsider what treatments currently work best, and what areas to focus on going forward. While developing serotonergic or GABAergic agents with more favorable side effect profiles (compared to SSRIs, SNRIs, and benzodiazepines, gabapentin and pregabalin) may have some clinical value, there needs to be further expansion into agents targeting neuropeptide pathways, glutamate, endocannabinoids, and multi-modal medications (including phytochemicals and hallucinogens). These newer compounds may not replace the current treatments but may over time serve as adjuncts or aid in therapy, at least until the field can develop better biomarkers and incorporate brain imaging, pharmacogenomic and other neurobiochemical advances. In terms of pharmacological development, it is time for anxiety disorders to catch up to depression, PTSD, bipolar disorder, and schizophrenia.

## Author Contributions

AG, JM, RT, RF, and DI contributed to conception and design and wrote the original draft of the manuscript. AG, JM, RF, RT, KL, FB, and DI contributed to manuscript revisions, review and analysis of the literature, and creation of the tables. All authors reviewed and approved the final draft of the manuscript and made substantial contributions to this study.

## Conflict of Interest

In the past 5 years, JM has provided consultation services and/or served on advisory boards for Allergan, Boehreinger Ingelheim, Clexio Biosciences, Fortress Biotech, FSV7, Global Medical Education (GME), Impel Neuropharma, Janssen Research and Development, Medavante-Prophase, Novartis, Otsuka, and Sage Therapeutics. JM is named on a patent pending for neuropeptide Y as a treatment for mood and anxiety disorders and on a patent pending for the use of ezogabine and other KCNQ channel openers to treat depression and related conditions. The Icahn School of Medicine (employer of JM) is named on a patent and has entered into a licensing agreement and will receive payments related to the use of ketamine or esketamine for the treatment of depression. The Icahn School of Medicine is also named on a patent related to the use of ketamine for the treatment of PTSD. JM is not named on these patents and will not receive any payments. In the last 5 years, DI has received consulting honoraria from Alkermes, Axsome, Centers for Psychiatric Excellence, Jazz, Lundbeck, Otsuka, Precision Neuroscience, Sage, Sunovion; he has received research support (through his academic institution) from Alkermes, Astra Zeneca, Brainsway, Litecure, Neosync, Otsuka, Roche, Shire. The remaining authors declare that the research was conducted in the absence of any commercial or financial relationships that could be construed as a potential conflict of interest.
